# Accurate Prediction of Humphrey 10-2 Visual Fields in Glaucoma from a Single Rapid IMOvifa 24plus(1-2)

**DOI:** 10.1016/j.xops.2026.101127

**Published:** 2026-02-20

**Authors:** Yuka Igari, Euido Nishijima, Kei Sano, Aiko Iwase, Tadashi Nakano

**Affiliations:** 1 Department of Ophthalmology, The Jikei University School of Medicine, Tokyo, Japan; 2 Tajimi Iwase Eye Clinic, Tajimi, Japan

**Keywords:** Central visual field, Glaucoma, IMOvifa, Machine learning

## Abstract

**Purpose:**

Predicting the Humphrey Field Analyzer (HFA) 10-2 visual field (VF) using machine learning (ML) based on IMOvifa 24plus(1-2) VF data.

**Design:**

Retrospective cross-sectional study.

**Participants:**

Seventy actual IMOvifa 24plus(1-2) tests from 25 patients (The Jikei University School of Medicine) and 3472 synthesized IMOvifa 24plus(1-2) tests from 884 patients who underwent HFA 24-2 and HFA 10-2 VF measurements at 4 affiliated hospitals.

**Methods:**

Synthesized 24plus(1-2) data were created by merging 54 points from HFA 24-2 and 24 points from HFA 10-2 tests. An XGBoost model, trained on the synthetic data set, predicted thresholds at the 68 HFA 10-2 test locations. Model performance was assessed on the actual data set using leave-one-out cross-validation. Stratified patient-level bootstrap analyses accounting for multiple tests per patient were used to compare results across 3 glaucoma severity groups (mild, moderate, and advanced) based on HFA 10-2 mean deviation. Four models utilizing different input subsets were evaluated: model 1 (all 78 IMOvifa 24plus[1-2] points), model 2 (54 points of 24-2), model 3 (central 40 points of 24plus[1-2]), and model 4 (central 16 points of 24-2). Test durations were also compared.

**Main Outcome Measures:**

Mean absolute error (MAE), root mean squared error (RMSE), and coefficient of determination (R^2^) between predicted and measured HFA 10-2 sensitivities.

**Results:**

Model 1 (all 24plus[1-2] points) achieved the highest overall accuracy (MAE 3.59 dB, RMSE 5.71 dB, R^2^ 0.76), significantly outperforming model 2 (24-2 points; MAE 4.15 dB, RMSE 6.31 dB, R^2^ 0.70) (*P* < 0.05 for all metrics). Similarly, model 3 significantly outperformed model 4 (*P* < 0.05). Stratified analysis indicated that adding central test points yielded significant accuracy improvements, consistently across all metrics in the moderate group, whereas results varied by metric in the mild and advanced groups. The IMOvifa 24plus(1-2) test duration (mean 155 seconds) was significantly shorter than the HFA 10-2 Swedish Interactive Thresholding Algorithm Standard test (mean 376 seconds) (*P* < 0.001).

**Conclusions:**

Incorporating the additional central test points from the IMOvifa 24plus(1-2) significantly enhances the accuracy of ML based HFA 10-2 VF prediction. This approach offers an efficient strategy for obtaining detailed central VF information from a single, rapid test, potentially improving glaucoma management.

**Financial Disclosure(s):**

Proprietary or commercial disclosure may be found in the Footnotes and Disclosures at the end of this article.

Glaucoma is a leading cause of blindness worldwide, and the number of patients is expected to increase to 112 million by 2040.^1^, ^2^, ^3^, ^4^ Glaucoma causes progressive retinal ganglion cell death and associated visual field (VF) defects that can eventually lead to blindness if not treated appropriately. Visual field testing is the primary tool for detecting glaucoma and monitoring its progression. At present, the Humphrey Field Analyzer (HFA) (Carl Zeiss Meditec, Inc.) 24-2 VF is the most widely used form of perimetry in the diagnosis and follow-up of glaucoma.^5^, ^6^, ^7^, ^8^

Glaucoma typically presents with peripheral VF defects; however, central VF defects (CVFDs) are also frequently observed, even in mild-stage glaucoma.^9^ Several studies have indicated that assessing 12 test points within the central 10° of the HFA 24-2 is comparable in effectiveness to the HFA 10-2 for detecting CVFDs.^6^^,^^7^ Conversely, the HFA 24-2 may often fail to identify VF defects within the central 10°.^10^

A previous study reported that 16 of 26 eyes (61.5%) classified as normal based on the HFA 24-2 in the glaucoma group were found to be abnormal on HFA 10-2.^11^ Therefore, while performing both HFA 24-2 and HFA 10-2 examinations is optimal, particularly for evaluating the central 10° VF, the implementation of both protocols for all patients with glaucoma and glaucoma suspects is often impractical due to the considerable burden it places on patients, health care resources, and staff.

Recently, the 24-2C Swedish Interactive Thresholding Algorithm (SITA) Faster and the 24plus(1-2) program for IMOvifa perimetry (CREWT Medical Systems) have been introduced. Both programs add test points within the central 10° to the conventional HFA 24-2 pattern.^12^^,^^13^ The IMOvifa does not require a darkroom, permits binocular testing, and has demonstrated comparable accuracy to the HFA.^14^ The IMOvifa 24plus(1-2) incorporates 24 test points from the HFA 10-2 into the HFA 24-2. The addition of these test points within the central 10° has been reported to enhance the assessment of the central VF.^12^ However, it does not encompass all test points of the HFA 10-2 SITA Standard. Therefore, the ability to reliably generate a “virtual HFA 10-2” from IMOvifa 24plus(1-2) data could potentially provide Supplementary Information (available at www.ophthalmologyscience.org) about the central VF without requiring additional HFA 10-2 testing. Specifically, in mild to moderate stage glaucoma cases, even if the IMOvifa 24plus(1-2) reveals only subtle abnormalities within the central 10°, a model predicting detailed CVFDs could allow clinicians to anticipate findings from an actual HFA 10-2 and plan follow-up accordingly.^15^

Recent research has focused on estimating and reproducing VF test results using machine learning (ML) and deep learning techniques.^16^, ^17^, ^18^, ^19^, ^20^ For instance, Sugisaki et al^16^ employed ML to predict HFA 10-2 outcomes from HFA 24-2 data in advanced glaucoma, demonstrating a reduction in mean absolute error (MAE) at each test point to approximately 4 dB. Furthermore, other studies have reported improved accuracy by incorporating OCT data, such as retinal nerve fiber layer (RNFL) thickness around the optic disc and macular thickness maps, as input features.^17^, ^18^, ^19^, ^20^ We hypothesized that a VF test incorporating additional central test points, such as the IMOvifa 24plus(1-2) protocol, would yield more accurate predictions of the central 10° VF. Specifically, we posited that the enhanced central point density of the IMOvifa 24plus(1-2) would improve prediction accuracy compared to using only the standard HFA 24-2 points within the central 10°. In this study, we employed an ML model to predict HFA 10-2 results under 4 different input configurations: using all measurement points from the IMOvifa 24plus(1-2), using all measurement points from the HFA 24-2, using only the measurement points within the central 10° of the IMOvifa 24plus(1-2), and using only the measurement points within the central 10° of the HFA 24-2. Furthermore, we compared the testing time between the IMOvifa 24plus(1-2) and the HFA 10-2 SITA Standard tests.

## Methods

### Informed Consent and Ethical Approval

Written informed consent was obtained from all participants before enrollment. All procedures were conducted in accordance with the ethical principles outlined in the Declaration of Helsinki for medical research involving human subjects and received approval from the ethics committee of The Jikei University School of Medicine (approval no.: 26-3[7879]).

### Study Design and Participant Selection

This retrospective study recruited patients with glaucoma or suspected glaucoma at The Jikei University School of Medicine from February 2022 to March 2024.

The inclusion criteria for this study were as follows: (1) patients with glaucoma or suspected glaucoma who were regularly followed up at The Jikei University School of Medicine, irrespective of treatment status; (2) patients who had previously undergone HFA 24-2 SITA Standard VF testing, with mean deviations (MDs) of ≥ –25 dB; (3) best-corrected visual acuity (BCVA), expressed as the logarithm of the minimum angle of resolution, of ≤ 0.3 (equivalent to Snellen 20/40 or better); (4) patients aged 20 years or older, for whom normative reference data for the general population are available. Sex was not an inclusion criterion. The exclusion criteria were as follows: (1) patients who did not provide informed consent; (2) eyes with ocular findings other than glaucoma that could affect the VF; (3) eyes with a history of intraocular surgery other than cataract or microinvasive glaucoma surgery; (4) cases that did not meet the reliability criteria for the VF tests (mentioned in subsequent sections).

### Ophthalmic Examination and Glaucoma Diagnosis

Each patient underwent a comprehensive ophthalmic examination for glaucoma diagnosis, which included the measurement of BCVA, the measurement of intraocular pressure (IOP) by Goldmann applanation tonometry, slit lamp biomicroscopy, gonioscopy, corneal pachymetry, dilated ophthalmoscopy, and fundus photography. Glaucoma and suspected glaucoma were diagnosed by glaucoma specialists (Y. I., E. N., K. S., and T. N.), as previously described, based on the presence of glaucomatous optic neuropathy and corresponding VF defects. Glaucomatous optic neuropathy was diagnosed according to the following criteria: (1) a vertical cup-to-disc ratio of ≥ 0.7 at the optic nerve head; (2) a rim-to-disc ratio of ≤ 0.1 in the superior (11–1 o'clock) or inferior (5–7 o'clock) regions; (3) a difference in the vertical cup-to-disc ratios between the left and right eyes of ≥ 0.2; or 4) the presence of an RNFL defect. A glaucomatous VF defect was diagnosed using HFA 24-2 SITA Standard according to the following criteria: (1) the glaucoma hemifield test results were outside normal limits; (2) the pattern deviation probability plots in either the upper or lower hemifield showed a cluster of 3 or more contiguous nonedge points with sensitivities of < 5%, at least one of which had a probability of < 1%; or (3) the pattern standard deviation (PSD) was significant (*P* < 5%).

### Imo Perimetry

The IMOvifa is the successor to the imo, a portable head-mounted perimeter also from CREWT Medical Systems.^21^ While capable of testing each eye independently, the IMOvifa can also present stimuli randomly to either eye in a nonoccluded setting, without the examinee's awareness of the tested eye.^22^ All patients underwent both examinations (IMOvifa 24plus[1-2] and HFA 10-2 SITA Standard) in a randomized order, with each examination performed within a 6-month period for each patient. Reliability criteria were defined as fixation losses less than 25%, a false-positive response rate <15%, and a false-negative response rate <20%.^23^

IMOvifa uses Ambient Interactive ZEST, a proprietary measurement strategy that employs Bayesian inference and maximum likelihood methods for threshold determination. Ambient Interactive ZEST has been shown to reduce test duration by approximately 70% compared to the 4-2 dB bracketing technique.^22^ Ambient Interactive ZEST-Rapid builds upon the Ambient Interactive ZEST method, incorporating a stronger emphasis on interactions between adjacent measurement points and reducing the number of stimulus presentations by estimating the reliability index. This achieves a faster testing time while preserving accuracy.^13^^,^^24^

### Data Augmentation for Synthesizing IMOvifa 24plus(1-2)

The 78 points in the IMOvifa 24plus(1-2) correspond to measurement locations in the HFA 24-2 and HFA 10-2 tests. The synthesized IMOvifa 24plus(1-2) (78 points) was generated by combining pointwise sensitivities from HFA 24-2 (54 points) and HFA 10-2 (additional 24 points located within the central 10 degrees), derived from paired data sets conducted within a 6-month window.^25^ This synthesis process incorporated HFA tests performed between October 14, 2008, and October 5, 2023, at 4 hospitals affiliated with The Jikei University School of Medicine.

### Model Development and Evaluation

**Figure 1:**
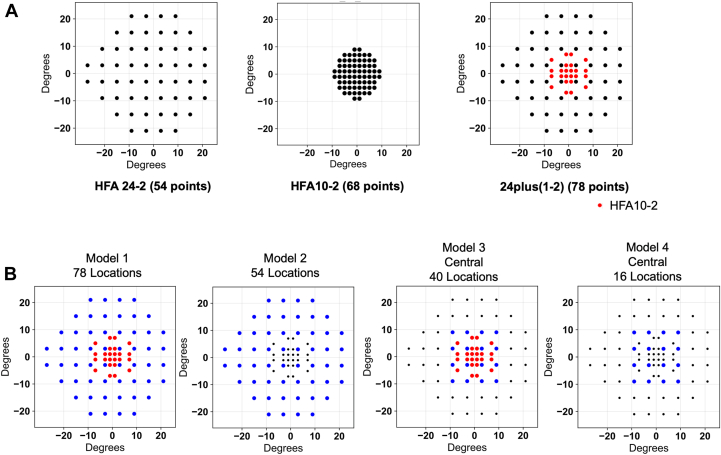
Spatial arrangement of test point locations and definition of input models. (**A)** Test points of the HFA 24-2 (54 points, left), 10-2 (68 points, middle), and the IMOvifa 24plus(1-2) (78 points, right). For the 24plus(1-2) pattern, black dots represent standard 24-2 locations, while red dots indicate additional central locations corresponding to the 10-2. (**B)** Four input models using subsets of 24plus(1-2) points: model 1 (all 78 IMOvifa 24plus(1-2) points), model 2 (54 points of 24-2), model 3 (central 40 points of 24plus[1-2]), and model 4 (central 16 points of 24-2). HFA = Humphrey Field Analyzer.

In this study, we used XGBoost to predict the pointwise sensitivities at the 68 points of the HFA 10-2 using subsets of the 78 points from the IMOvifa 24plus(1-2) test and patient age as input features. For the input data, separate models were developed for each subset of test points (along with age), defined as model 1, model 2, model 3, and model 4 (Fig 1). Considering the stochastic variability introduced by random seed dependency, seed randomization was applied using 10 different seeds for each combination of modeling architecture and input data subset. To prevent information leakage from data clustering (i.e., repeated measures from the same participant), the model's performance was rigorously evaluated using a strict leave-one-out cross-validation that ensured patient-level independence. This approach involved 70 independent training and evaluation cycles. In each cycle, one test was held out from the 70 actual tests as the validation set. Subsequently, all other records belonging to the same patient as the held-out test (e.g., tests from the contralateral eye or other visits) were completely excluded from the remaining set of actual tests. A training data set was then constructed by combining this patient-excluded set of actual tests with the entire synthesized data set (N = 3472), and the model was retrained from scratch. The model's performance was evaluated solely on the held-out validation test. This process was repeated 70 times, and the final performance metrics were calculated by aggregating the predictions from all cycles.

**Figure 2:**
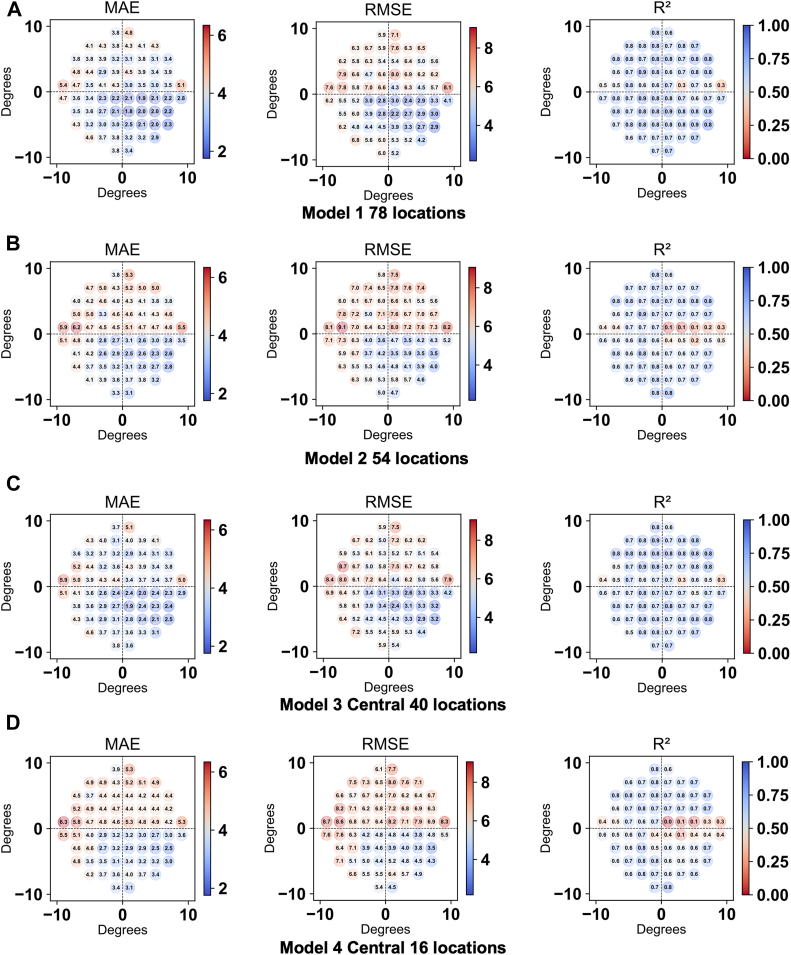
Pointwise performance metrics for HFA 10-2 prediction using different input point sets from the IMOvifa 24plus(1-2) test. Heatmaps show performance across the 10-2 grid for each model defined in Figure 1: **(A)** model 1 (78 locations), **(B)** model 2 (54 locations), **(C)** model 3 (central 40 locations), and **(D)** model 4 (central 16 locations). MAE = mean absolute error; RMSE = root mean squared error; R^2^ = coefficient of determination.

For each of the 68 test points of the HFA 10-2, we calculated the MAE, root mean squared error (RMSE), and a measure of correlation (coefficient of determination [R^2^]) to assess the agreement between predicted and measured threshold values. Lower values for MAE and RMSE reflect greater predictive accuracy, whereas higher R^2^ values suggest superior model performance. To account for clustering arising from multiple tests per patient, we employed a patient-level bootstrap method (2000 iterations) to estimate these global performance metrics and their 95% confidence intervals (CIs). Specifically, bootstrap samples were generated by resampling patients (identifications) rather than individual test points. Comparisons between model 1 and model 2, and between model 3 and model 4, were performed using a paired patient-level bootstrap test. In each iteration, the difference in performance metrics (MAE, RMSE, and R^2^) between the paired models was calculated using the same set of resampled patients. Statistical significance was determined if the 95% CI of the difference did not include zero, and *P* values were derived from the bootstrap distribution. To prevent data leakage and ensure rigorous evaluation, we calculated the performance metrics after excluding the 24 test locations that overlap between IMOvifa 24plus(1-2) and HFA 10-2. Two different approaches were used to calculate R^2^ based on the analytical objective. To visualize spatial performance patterns (Fig 2), a pointwise R^2^ was computed for each of the 68 test points individually. To provide a single, robust metric for overall model comparison, an overall R^2^ was derived from the entire pooled data set of all points from all tests. In addition, we performed stratified analyses based on HFA 10-2 MD values, dividing the data into 3 groups: mild (MD ≥ –6 dB), moderate (–12 dB ≤ MD < –6 dB), and advanced (MD < –12 dB). Within each MD stratum, the performances of models 1 and 2, and models 3 and 4, were compared using the same paired patient-level bootstrap approach. Furthermore, we compared the test duration among IMOvifa 24plus(1-2), HFA 10-2, and their sum using the Tukey–Kramer post hoc test. In all analyses, a *P* value < 0.05 was considered statistically significant. All model development and statistical analyses were performed using Python (version 3.12.4, Python Software Foundation).

## Results

This retrospective study included 70 IMOvifa 24plus(1-2) examinations from 47 eyes of 25 patients with glaucoma or suspected glaucoma from The Jikei University School of Medicine as the testing data set. Additionally, 3472 synthesized IMOvifa 24plus(1-2) data sets were generated by combining paired HFA 24-2 and HFA 10-2 test results from 1568 eyes of 884 patients, serving as the training data set. Demographic and clinical characteristics of the training and testing data sets are summarized in Table 1. A significant difference was observed only in age between the 2 data sets (testing data set: mean age 60.2 ± 12.9 years; training data set: mean age 57.2 ± 9.89 years; *P* < 0.001). No significant differences were found in MD from either HFA 24-2 or HFA 10-2 tests, PSD from either HFA 24-2 or HFA 10-2 tests, VF Index, or glaucoma hemifield test results between the training and testing data sets (Table 1).

**Table 1:** Systematic Characteristics of Patients

Characteristic	Training Data Set	Testing Data Set	*P* Value
Number of patients (eyes)	884 (1568)	25 (47)	
Number of tests	3472	70	
Age (yrs)	57.2 ± 9.9	60.2 ± 12.9	<0.001
Right: left (tests)	1718: 1754	36: 34	
MD of 24-2 (dB)	–9.10 ± 8.41	–8.40 ± 7.40	0.73
MD of 10-2 (dB)	–8.60 ± 8.99	–6.73 ± 7.25	0.18
PSD of 24-2 (dB)	8.22 ± 4.65	8.90 ± 4.50	0.24
PSD of 10-2 (dB)	7.41 ± 5.17	7.45 ± 5.14	0.82
VFI (%)	73.3 ± 25.3	74.4 ± 21.4	0.99
GHT, N (%)	2798 (80.6%)	58 (82.9%)	0.63

GHT = glaucoma hemifield test; MD = mean deviation; PSD = pattern standard deviation; VFI = Visual Field Index.

Statistical significance is indicated (*P* < 0.05, 2-sided Mann–Whitney U test).

We developed ML models to predict the 68 pointwise sensitivity values of the HFA 10-2 VF test using specific subsets of test points from the IMOvifa 24plus(1-2) examination. Four distinct models (Model 1-4) were created based on different input configurations (Fig 1).

Comparisons were made between model 1 versus model 2 and model 3 versus model 4. The overall MAE values were 3.59 dB, 4.15 dB, 3.63 dB, and 4.25 dB for models 1, 2, 3, and 4, respectively. The overall RMSE values were 5.71 dB, 6.31 dB, 5.76 dB, and 6.49 dB for models 1, 2, 3, and 4, respectively. The overall R^2^ values were 0.76, 0.70, 0.79, and 0.68 for models 1, 2, 3, and 4, respectively. Model 1 consistently outperformed model 2, and model 3 consistently outperformed model 4 across all 3 metrics (MAE, RMSE, and R^2^) (Table 2). To evaluate the potential influence of overlapping test locations between IMOvifa 24plus(1-2) and HFA 10-2, we re-evaluated model performance after excluding the 24 test locations that overlapped between the 2 grids. In this analysis, MAE, RMSE, and R^2^ were recalculated using only the remaining nonoverlapping HFA 10-2 test points, and the results are presented together with the primary performance metrics in Table 2.

**Table 2:** Performance of 4 Machine Learning Models for HFA 10-2 VF Prediction, Stratified by Glaucoma Severity

	78 Locations (Model 1)	54 Locations (Model 2)	Central 40 Locations (Model 3)	Central 16 Locations (Model 4)	Mean Difference (95% CI) (1 vs. 2)	*P* Value (1 vs. 2)	Mean Difference (95% CI) (3 vs. 4)	*P* Value (3 vs. 4)
MAE (dB) (total)	3.59	4.15	3.63	4.25	–0.56 (–0.79 to –0.37)	<0.05	–0.62 (–0.96 to 0.33)	<0.05
MAE (dB) (–6 ≤ MD)	2.63	3.16	2.79	3.27	–0.52 (–0.89 to –0.19)	<0.05	–0.48 (–1.01 to 0.02)	0.06
MAE (dB) (–12 ≤ MD < –6)	4.96	5.17	5.26	5.93	–0.75 (–1.22 to –0.34)	<0.05	–0.67 (–1.20 to –0.18)	<0.05
MAE (dB) (MD < –12)	4.10	4.58	3.69	4.55	–0.48 (–0.89 to –0.23)	<0.05	–0.86 (–1.37 to –0.52)	<0.05
RMSE (dB) (total)	5.71	6.31	5.76	6.49	–0.60 (–1.06 to –0.25)	<0.05	–0.72 (–1.27 to –0.27)	<0.05
RMSE (dB) (–6 ≤ MD)	4.34	5.10	4.44	5.27	–0.76 (–1.52 to –0.01)	<0.05	–0.83 (–1.93 to 0.15)	0.11
RMSE (dB) (–12 ≤ MD < –6)	7.10	7.89	7.29	8.09	–0.79 (–1.38 to –0.34)	<0.05	–0.80 (–1.41 to –0.26)	<0.05
RMSE (dB) (MD < –12)	6.51	6.73	6.24	6.83	–0.22 (–0.74 to 0.13)	0.31	–0.59 (–1.30 to –0.16)	<0.05
R^2^ (total)	0.76	0.70	0.75	0.68	0.05 (0.02–0.11)	<0.05	0.07 (0.02–0.13)	<0.05
R^2^ (–6 ≤ MD)	0.46	0.26	0.43	0.22	0.19 (–0.00 to 0.38)	0.06	0.21 (–0.06 to 0.48)	0.12
R^2^ (–12 ≤ MD < –6)	0.55	0.45	0.52	0.42	0.11 (0.04–0.19)	<0.05	0.11 (0.03–0.19)	<0.05
R^2^ (MD < –12)	0.77	0.75	0.79	0.75	0.01 (–0.01 to 0.04)	0.33	0.04 (0.01–0.08)	<0.05

CI = confidence interval; HFA = Humphrey Field Analyzer; MAE = mean absolute error; MD = mean deviation; RMSE = root mean squared error; R^2^ = Coefficient of determination; VF = visual field.

*P* values and 95% confidence intervals were estimated using paired patient-level bootstrap resampling (resampling unit: patient ID, 2000 iterations) to account for clustering. To prevent data leakage, performance metrics (MAE, RMSE, and R^2^) for all models were calculated using the 44 nonoverlapping test locations of the HFA 10-2.

The predictive performance was further analyzed by stratifying the data into 3 groups based on HFA 10-2 MD values: mild (–6 dB ≤ MD), moderate (–12 dB ≤ MD < –6 dB), and severe (MD < –12 dB). In the mild group, model 1 (MAE: 2.63 dB, RMSE: 4.34 dB) demonstrated significantly lower prediction errors compared with model 2 (MAE: 3.16 dB, RMSE: 5.10 dB; *P* < 0.05 for both), although the difference in R^2^ did not reach statistical significance (0.46 vs. 0.26; *P* = 0.06). No statistically significant differences were observed between model 3 and model 4 across any metrics in the mild group (*P* ≥ 0.06). In the moderate group, the inclusion of additional central points yielded consistent improvements. Model 1 significantly outperformed model 2 across all metrics (MAE: 4.96 vs. 5.17 dB; RMSE: 7.10 vs. 7.89 dB; R^2^: 0.55 vs. 0.45; *P* < 0.05 for all). Similarly, model 3 showed significantly superior performance compared with model 4 (MAE: 5.26 vs. 5.93 dB; RMSE: 7.29 vs. 8.09 dB; R^2^: 0.52 vs. 0.42; *P* < 0.05 for all). In the severe group, model 1 yielded a significantly lower MAE compared with model 2 (4.10 vs. 4.58 dB; *P* < 0.05); however, differences in RMSE (6.51 vs. 6.73 dB; *P* = 0.31) and R^2^ (0.77 vs. 0.75; *P* = 0.33) were not statistically significant. In contrast, model 3 demonstrated significantly better performance than model 4 across all metrics: MAE (3.69 vs. 4.55 dB), RMSE (6.24 vs. 6.83 dB), and R^2^ (0.79 vs. 0.75) (*P* < 0.05 for all comparisons) (Table 2).

**Figure 3:**
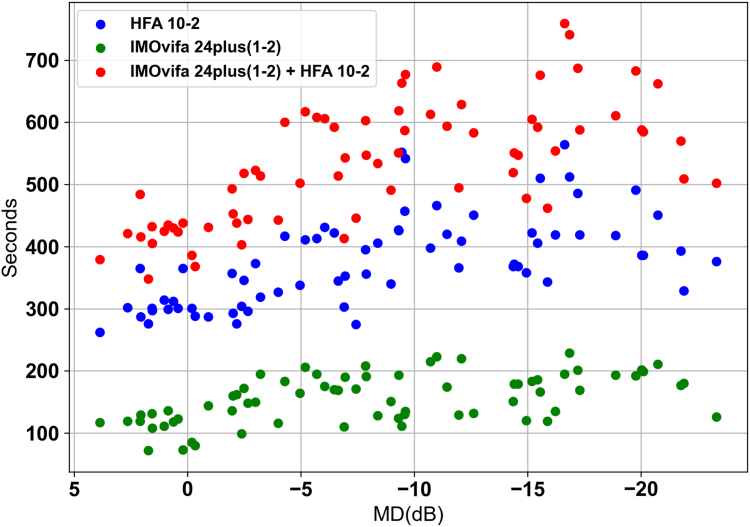
Scatter plot of MD values and measurement times for each test. Test times of the IMOvifa 24plus(1-2) (green), HFA 10-2 (blue), and their sum (red) at each MD value are plotted. HFA = Humphrey Field Analyzer; MD = mean deviation.

The test duration for the IMOvifa 24plus(1-2) was 155 seconds (95% CI: 146–165 seconds). The test duration for the HFA 10-2 SITA Standard test was 376 seconds (95% CI: 359–394 seconds). The IMOvifa 24plus(1-2) required significantly less time to complete compared to the HFA 10-2 SITA Standard (*P* < 0.001) (Fig 3, Table 3).

**Table 3:** Testing Time of Each Program

	Testing Time (sec)	*P* Value
IMOvifa 24plus(1-2)	155 (146–165)	<0.001∗, <0.001†
HFA 10-2	376 (359–394)	
IMOvifa 24plus(1-2) + HFA 10-2	532 (509–555)	

HFA = Humphrey Field Analyzer.

Tukey–Kramer post hoc test.

∗ Compared to HFA 10-2.

† Compared to IMOvifa 24plus(1-2) + HFA 10-2.

## Discussion

The primary objective of this study was to determine if VF tests incorporating additional central points, such as the IMOvifa 24plus(1-2), enable accurate prediction of HFA 10-2 results using ML. To address this, we developed and evaluated 4 ML models (models 1-4) utilizing different input data subsets derived from the 24plus(1-2) test grid. We employed XGBoost and assessed their predictive performance using leave-one-out cross-validation.

Our findings indicate that among the 4 ML models evaluated, model 1, which utilized all 78 test points from the IMOvifa 24plus(1-2) as input data, demonstrated the highest accuracy in predicting HFA 10-2 pointwise sensitivities. Specifically, ML models incorporating the additional central 10-degree test points from the IMOvifa 24plus(1-2) (models 1 and 3) achieved statistically significantly higher predictive accuracy compared to models relying solely on the HFA 24-2 equivalent points (models 2 and 4). This improvement in accuracy was particularly pronounced in eyes with mild and moderate glaucoma, with a tendency for greater improvement observed especially in the superior VF. Furthermore, the testing time for the IMOvifa 24plus(1-2) was significantly shorter compared to the HFA 10-2 SITA Standard (mean 155 seconds vs. 376 seconds), demonstrating its efficiency.

Previous studies have also reported that utilizing VF tests with added points within the central 10° can improve the detection capability for CVFDs. Montesano et al^26^ showed that adding test points within the central 10° slightly improved the detection sensitivity for CVFDs without significant loss of specificity. Furthermore, Ehrlich et al^27^ demonstrated through simulation that adding HFA 10-2-derived points within the central 10° of the standard HFA 24-2 significantly improved the detection accuracy (sensitivity and specificity) for early glaucomatous CVFDs. The finding that predictive accuracy decreased as MD worsened is consistent with previous reports, likely because greater severity involves larger variability in CVFDs, making the prediction task more challenging.^25^ While these prior studies suggested the potential for improved detection by adding central points, our study quantitatively demonstrates that using an ML approach for central VF prediction, the additional central points in the IMOvifa 24plus(1-2) significantly improve the overall HFA 10-2 prediction accuracy.

An interesting and consistent trend was observed in the accuracy of pointwise HFA 10-2 prediction between the current study and 3 previous studies utilizing models derived from RNFL thickness or macular measurement data.^20^^,^^28^^,^^29^ Mean absolute error tended to be lower in the inferotemporal locations of the HFA 10-2. This area corresponds to the “central isle” previously described by Weber et al^30^ as a region of VF preservation in patients with glaucoma. The concordance between results from data-driven models based on optic nerve head, macular regions, and now the IMOvifa 24plus(1-2), along with previous clinical descriptions, further supports the view that glaucomatous VF loss follows characteristic patterns that relatively spare the inferotemporal region of the HFA 10-2. The lower MAE in this region is likely attributable to the relatively preserved VF sensitivity and smaller variability of test points in this area.

Furthermore, and importantly, as illustrated in Figure 2, model 1 showed particularly notable improvement in MAE and RMSE compared to model 2 in the prediction of the superior VF of the HFA 10-2 test. The enhanced prediction accuracy in the superior field is likely related to both the pathophysiology of glaucoma and the learning characteristics of ML models. Clinically, early glaucomatous VF changes, such as paracentral or arcuate scotomas, frequently manifest in the superior nasal and corresponding superior temporal areas, that is, the superior VF.^31^ Indeed, a post hoc analysis of our training data set confirmed a significant asymmetry in VF defects, with the mean threshold sensitivity in the superior hemifield being significantly lower than in the inferior hemifield (21.9 ± 10.5 dB vs. 26.1 ± 9.11 dB, respectively; *P* < 0.05). Since ML models tend to learn frequently occurring patterns more effectively, this prevalence of superior defects in the training data likely explains the enhanced predictive performance observed in the superior VF. Model 1 directly incorporates sensitivity information from 12 points of the HFA 10-2 grid located within this superior central region where glaucomatous changes frequently occur. This likely allowed the model to preferentially learn the frequent superior CVFD patterns within the data set, enhancing its ability to capture these features. Consequently, model 1 acquired an improved capability to predict sensitivity loss in the superior VF, contributing to the observed enhancement in prediction accuracy in this region.

Notably, model 3 with only 40 input points outperformed model 2 with 54 points, suggesting that efficient prediction can be achieved when fewer input points capture the most relevant features. This advantage of model 3 may be attributed to its input configuration including 24 points aligned exactly with the 10-2 grid plus 16 nearby points, all of which were highly pertinent to the prediction target. In contrast, inferior performance of model 2 is plausible, since selecting features irrelevant to the prediction target generally undermines learning efficiency and overall model capacity.^32^

Another noteworthy finding is the variation in model performance across glaucoma severities. The predictive accuracy (R^2^) was lower in the mild glaucoma group (R^2^: –0.46) compared to the moderate (R^2^: 0.55) and severe (R^2^: 0.77) groups. This phenomenon is best explained by the signal-to-noise ratio. In early-stage disease, true VF defects (the “signal”) are shallow and subtle, making them difficult to distinguish from the inherent perimetric test–retest variability (the “noise”). As glaucoma progresses, the defects become deeper and more extensive, providing a strong, unambiguous signal that allows for a more robust and reliable prediction. This finding suggests that the model may not be suitable as a standalone tool for definitively diagnosing central defects in early-stage patients. Instead, its primary utility is as a risk stratification and decision-support tool, helping clinicians identify patients at a higher risk of central VF loss who should be prioritized for the HFA 10-2.

Regarding test duration, the IMOvifa 24plus(1-2) required significantly less time compared to the HFA 10-2 SITA Standard (mean 155 seconds vs. 376 seconds), despite having more test points. Moreover, the IMOvifa allows for testing with both eyes open and does not require a darkroom, which can reduce patient discomfort and feelings of confinement associated with traditional perimetry. These features can alleviate the burden on both patients and testing staff, improving the feasibility of VF testing in routine clinical practice.

This study has several limitations. First, the results are based on data from specific institutions and a limited patient background; thus, external validity has not been verified. Therefore, confirming the broad clinical utility of this model will require prospective validation across diverse patient populations (different ethnicities, glaucoma severities, comorbidities) and multiple institutions. Second, the number of actual IMOvifa 24plus(1-2) tests available for training was limited, although this was mitigated by data augmentation using synthesized data sets. Indeed, leveraging synthesized data, as demonstrated in other VF research contexts, offers a practical pathway to develop robust models even when specific test data are not abundant, thereby promoting scalability in model development.^25^ Third, we did not compare the potential benefits of adding structural parameters (e.g., OCT measurements), IOP, or refractive error data. Fourth, the predictive performance of our model varied with disease severity. In mild glaucoma, while the absolute error (MAE: 2.63 dB) was low and comparable to test–retest variability,^33^ the relative performance metric (R^2^) was relatively low. This is likely because the minimal variance in near-normal VFs is difficult for the model to explain, suggesting a limited ability to capture very subtle defects within this subgroup. Conversely, as the disease progressed to moderate and severe group, the absolute prediction error increased, indicating that further improvements are needed for reliable application in patients with more severe VF loss.

In conclusion, this study demonstrates that utilizing ML with VF data incorporating additional central test points, such as those in the IMOvifa 24plus(1-2) protocol, significantly enhances the accuracy of predicting HFA 10-2 VFs. The ability to generate reliable “virtual HFA 10-2” information from a single, rapid, and patient-friendly IMOvifa 24plus(1-2) test presents a practical and efficient strategy for comprehensive glaucoma assessment. This potentially reduces the necessity for separate HFA 10-2 examinations, thereby lessening the burden on both patients (reducing test time, visit frequency, and fatigue) and health care resources (optimizing staff time and equipment utilization). Ultimately, this methodology holds significant promise for improving the detection and monitoring of CVFDs, particularly in clinical scenarios where frequent HFA 10-2 testing is challenging, potentially facilitating earlier identification of progression and more timely clinical interventions in glaucoma management.

## References

[1] Stein J.D., Khawaja A.P., Weizer J.S. (2021). Glaucoma in adults-screening, diagnosis, and management: a review. JAMA.

[2] Chen P.P. (2003). Blindness in patients with treated open-angle glaucoma. Ophthalmology.

[3] Blomdahl S., Calissendorff B.M., Tengroth B., Wallin O. (1997). Blindness in glaucoma patients. Acta Ophthalmol.

[4] Tham Y.C., Li X., Wong T.Y. (2014). Global prevalence of glaucoma and projections of glaucoma burden through 2040: a systematic review and meta-analysis. Ophthalmology.

[5] Jung K.I., Ryu H.K., Hong K.H. (2021). Simultaneously performed combined 24-2 and 10-2 visual field tests in glaucoma. Sci Rep.

[6] West M.E., Sharpe G.P., Hutchison D.M. (2021). Value of 10-2 visual field testing in glaucoma patients with early 24-2 visual field loss. Ophthalmology.

[7] Wu Z., Medeiros F.A., Weinreb R.N., Zangwill L.M. (2018). Performance of the 10-2 and 24-2 visual field tests for detecting central visual field abnormalities in glaucoma. Am J Ophthalmol.

[8] Onyekaba N.A.E., Estrela T., Naithani R. (2023). Comparison of 10-2 and 24-2 perimetry to diagnose glaucoma using OCT as an independent reference standard. Ophthalmol Glaucoma.

[9] Hood D.C., Raza A.S., de Moraes C.G.V. (2013). Glaucomatous damage of the macula. Prog Retin Eye Res.

[10] Traynis I., De Moraes C.G., Raza A.S. (2014). Prevalence and nature of early glaucomatous defects in the central 10° of the visual field. JAMA Ophthalmol.

[11] De Moraes C.G., Hood D.C., Thenappan A. (2017). 24-2 visual fields miss central defects shown on 10-2 tests in glaucoma suspects, ocular hypertensives, and early glaucoma. Ophthalmology.

[12] Nishijima E., Fukai K., Sano K. (2024). Comparative analysis of 24-2C, 24-2, and 10-2 visual field tests for detecting mild-stage glaucoma with central visual field defects. Am J Ophthalmol.

[13] Kimura T., Matsumoto C., Nomoto H. (2019). Comparison of head-mounted perimeter (imo) and humphrey field analyzer. Clin Ophthalmol (Auckl).

[14] Nishida T., Weinreb R.N., Arias J. (2023). Comparison of the TEMPO binocular perimeter and Humphrey field analyzer. Sci Rep.

[15] (2024). Central visual field testing in early glaucoma: a report by the American Academy of Ophthalmology. Ophthalmology.

[16] Sugisaki K., Asaoka R., Inoue T. (2020). Predicting Humphrey 10-2 visual field from 24-2 visual field in eyes with advanced glaucoma. Br J Ophthalmol.

[17] Shi M., Lokhande A., Tian Y. (2024). Transformer-based deep learning prediction of 10-Degree humphrey visual field tests from 24-degree data. Transl Vis Sci Technol.

[18] Asano S., Asaoka R., Murata H. (2021). Predicting the central 10 degrees visual field in glaucoma by applying a deep learning algorithm to optical coherence tomography images. Sci Rep.

[19] Hashimoto Y., Kiwaki T., Sugiura H. (2021). Predicting 10-2 visual field from optical coherence tomography in glaucoma using deep learning corrected with 24-2/30-2 visual field. Transl Vis Sci Technol.

[20] Kamalipour A., Moghimi S., Khosravi P. (2023). Deep learning estimation of 10-2 visual field map based on circumpapillary retinal nerve fiber layer thickness measurements. Am J Ophthalmol.

[21] Nishida T., Eslani M., Weinreb R.N. (2023). Perimetric comparison between the IMOvifa and Humphrey field analyzer. J Glaucoma.

[22] Matsumoto C., Yamao S., Nomoto H. (2016). Visual field testing with head-mounted perimeter “imo”. PLoS One.

[23] Kang J., Nascimento E., Silva R. (2025). Comparison of structure-function correlation among IMO visual function analyser and Humphrey field analyser. Br J Ophthalmol.

[24] Nomoto H., Matsumoto C., Okuyama S. (2023). A new static visual field test algorithm: the Ambient Interactive ZEST (AIZE). Sci Rep.

[25] Sano K., Nishijima E., Sumi S. (2025). Deep learning-based prediction of glaucoma severity and progression using Imo/TEMPO screening program. Ophthalmol Sci.

[26] Montesano G., McKendrick A.M., Turpin A. (2021). Do additional testing locations improve the detection of macular perimetric defects in glaucoma?. Ophthalmology.

[27] Ehrlich A.C., Raza A.S., Ritch R., Hood D.C. (2014). Modifying the conventional visual field test pattern to improve the detection of early glaucomatous defects in the central 10°. Transl Vis Sci Technol.

[28] Xu L., Asaoka R., Kiwaki T. (2020). Predicting the glaucomatous central 10-degree visual field from optical coherence tomography using deep learning and tensor regression. Am J Ophthalmol.

[29] Hashimoto Y., Asaoka R., Kiwaki T. (2021). Deep learning model to predict visual field in central 10° from optical coherence tomography measurement in glaucoma. Br J Ophthalmol.

[30] Weber J., Schultze T., Ulrich H. (1989). The visual field in advanced glaucoma. Int Ophthalmol.

[31] Hood D.C., Nguyen M., Ehrlich A.C. (2014). A test of a model of glaucomatous damage of the macula with high-density perimetry: implications for the locations of visual field test points. Translational Vis Sci Technol.

[32] Guyon I., Elisseeff A. (2003). An introduction to variable and feature selection. J Mach Learn Res.

[33] Wen J.C., Lee C.S., Keane P.A. (2019). Forecasting future Humphrey Visual Fields using deep learning. PLoS One.

