# Supplementary figures and images for: Accurate Prediction of Humphrey 10-2 Visual Fields in Glaucoma from a Single Rapid IMOvifa 24plus(1-2)
Source: Ophthalmol Sci. 2026 Feb 20;6(5):101127. doi: 10.1016/j.xops.2026.101127 (PMC13084673; doi:10.1016/j.xops.2026.101127)

## Supplemental Figure 1. Boxplots of pointwise performance metrics for HFA 10-2 prediction.

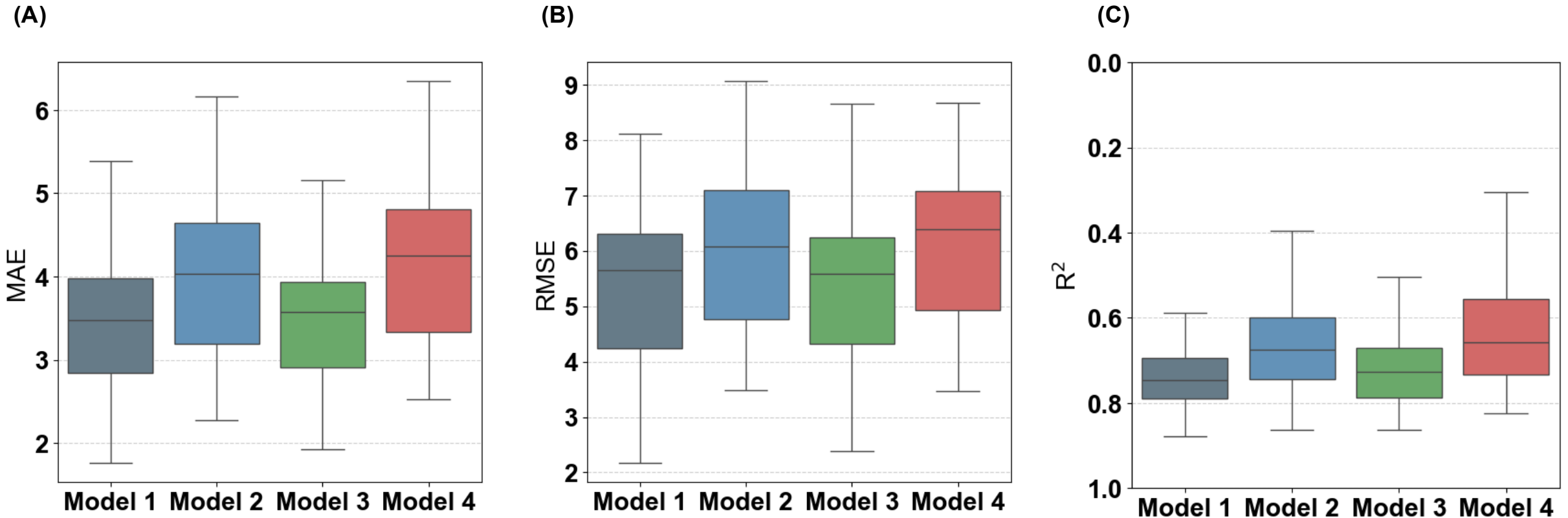

Supplement: Figure S1 [file mmc1.pdf]
